# The diagnostic value of Hsp90α in monitoring treatment responses in lung cancer

**DOI:** 10.55730/1300-0144.5369

**Published:** 2022-02-05

**Authors:** Min WANG, Zifeng JIANG, Rongyu LIU

**Affiliations:** 1 Department of Respiratory and Critical Care, the First Affiliated Hospital of Anhui Medical University, Anhui Province, China; 2 Department of Respiratory Medicine, Anhui Medical University Clinical College of Chest, Anhui Province, China

**Keywords:** Heat shock protein 90α, CYFRA21-1, CEA, NSE, lung cancer

## Abstract

**Background/aim:**

Heat shock protein 90α (Hsp90α) is considered a tumor biomarker in many human malignancies. This study investigated the diagnostic value of Hsp90α combined with other traditional lung cancer biomarkers (CEA, CYFRA21-1, and NSE) and its role in monitoring the treatment response of lung cancer patients.

**Materials and methods:**

A total of 205 patients with lung cancer and 186 patients with lung benign disease who were admitted to our hospital were enrolled from 2018 to 2020. The 205 patients included 76 cases of squamous, 92 cases of adenocarcinoma, and 37 cases of small cell lung cancer. There were 49 patients with TNM I+II and 156 patients with TNM III+IV. A total of 10 mL baseline peripheral venous blood samples and subsequent peripheral venous blood samples (7 days after two cycles of chemotherapy) were collected, and the levels of Hsp90α, carcinoembryonic antigen (CEA), Cytokeratin 19 fragments (CYFRA21-1), and neuron-specific enolase (NSE) were detected by ELISA kit.

**Results:**

Hsp90α was obviously higher in serum from patients with lung cancer than in patients with benign lung disease (p < 0.0001). Moreover, Hsp90α levels were higher in patients with advanced-stage (stage III–IV) lung cancer compared to those with early-stage (stage I–II). Hsp90α level was significantly decreased following treatment with chemotherapy in the progress partial response group (p = 0.017), whereas the level of Hsp90α was significantly higher after chemotherapy treatment in the progressive disease group (p < 0.0001). In addition, compared with CYFRA21-1, CEA, or NSE alone, the AUC of Hsp90α combined with CYFRA21-1, CEA, or NSE were significantly higher in the diagnosis of adenocarcinoma or small-cell lung cancer.

**Conclusion:**

Hsp90α combined with CYFRA21-1, CEA, and NSE can be used as diagnostic indicators of lung cancer. The Hsp90α level can be used to monitor treatment response.

## 1. Introduction

Lung cancer is the most common cancer and the main cause of cancer death worldwide. There were about 1.76 million cancer-related deaths worldwide in 2018 [1]. Although some progress has been made in immunotherapy, molecular targeted therapy, chemotherapy, radiotherapy and surgical treatment, the 5-year survival rate of lung cancer is significantly low [2]. Most patients with lung cancer are diagnosed in an advanced stage, and the prognosis is poor. This calls for the development of biomarkers to facilitate the early diagnosis of lung cancer.

Traditional biomarkers of lung cancer include carcinoembryonic antigen (CEA), cytokeratin 19 fragments (CYFRA21-1), and neuron-specific enolase (NSE). Among them, CEA was first reported in gastrointestinal carcinoma in 1965 and it has since been found to be upregulated in colorectal and lung cancer [3]. CYFRA21-1 and NSE were expressed in lung cancer and could be biomarkers of small cell lung cancer [4,5]. Carcinoma diagnosis needs histopathological confirmation. CEA, NSE, CYFRA21-1, and Hsp90α were tumor markers, which can be used as diagnostic tools. However, in order to improve the diagnostic efficiency, multiple tumor markers are usually detected and analyzed at the same time [3–5].

Heat shock proteins (HSPs) are a group of proteins that respond to extreme temperature changes, infection, inflammation, and hypoxia [6]. Hsp90, which was named based on its molecular weight, has two isoforms, Hsp90α and Hsp90β [7]. Hsp90α has been reported to be involved in the occurrence, development, and metastasis of tumors. This indicates that Hsp90α could be used as a marker for early detection of malignancy tumors [8]. However, the plasma Hsp90α level in lung cancer has rarely been reported.

Here, we measured the serum Hsp90α level in lung cancer patients and investigated the diagnostic value of Hsp90α combined with other traditional lung cancer biomarkers (CEA, CYFRA21-1, and NSE) and its role in monitoring the treatment response of lung cancer patients.

## 2. Materials

### 2.1. Participants

A total of 205 lung cancer patients and 186 benign lung disease patients treated in the First Affiliated Hospital of Anhui Medical University from 2018 to 2020 were enrolled in this study. The lung cancer was confirmed by the pathologic data of broncho-fiberscope or CT-guided lung biopsy. Evaluation of lung cancer patients was performed by improved tumor lymph node metastasis (TNM) classification [9]. Lung benign disease included pneumonia, COPD, etc. The patients were selected according to the following criteria: (a) primary lung cancer, (b) previously untreated, and surgery. Moreover, patients whose cancer arose in other sites were excluded.

The study protocol was approved by the Medical Ethics and Human Clinical Trial Committee of Anhui Medical University. All procedures in this study involving participants conform to the ethical standards of the agency and/or the National Research Council, as well as the 1964 Helsinki declaration and subsequent amendments or similar ethical standards. Informed consent was obtained from all individual participants.

In addition, a total of 127 advanced-stage lung cancer patients received chemotherapy, paclitaxel+carboplatin, pemetrexed+carboplatin, and etoposide+lobaplatin. Treatment efficacy was evaluated according to RECIST Version 1.1 (Response Evaluation Criteria in Solid Tumors) after the second cycle of chemotherapy [10]. Patients were grouped into partial response (PR), progressive disease (PD), and stable disease (SD) according to treatment response.

### 2.2. Detection of serum levels of Hsp90α, CEA, NSE, and CYFRA21-1

A total of 10 mL baseline peripheral venous blood samples and subsequent peripheral venous blood samples (7 days after two cycles of chemotherapy) were collected and were stored at −20 °C until use. The ELISA kits were used to measure the levels of HSP90α (#BMS2090, Invitrogen, USA), CEA (#A46341, Invitrogen, USA), NSE (E-EL-H1047c, Elabscience, China), and CYFRA21-1 (EH0364, FineTest, China). Briefly, the 96-well plate is preheated to 37 °C for 30 min. The standard and diluted plasma samples (1:50 dilution) were loaded into 96-well microplates. Gradually adding anti-Hsp90α HRP-conjugated antibodies and tetramethylbenzidine (TMB) substrate, the optical density (OD) was measured at 450 nm by spectrophotometer. The protein content of each sample was calculated according to the standard curve of OD value.

### 2.3. Statistical analyses

The SPSS 22.0 software (SPSS Inc., Chicago, IL, USA) was used for data analysis. The data were expressed as the median of abnormal distribution data (p_25_–p_75_). The correlation between Hsp90 concentration and pathological type was evaluated by the Kruskal Wallis test. Mann Whitney test was used to evaluate differences in Hsp90α level in relation to other clinicopathological variables, including gender (male vs. female), age (≥60 vs. < 60), TNM stage (I + II vs. III + IV), and so on. The receiver operating characteristic (ROC) curve was used to evaluate the diagnostic value of Hsp90α in lung cancer. An area under the ROC curve (AUC) value close to 1 represents good diagnostic accuracy, whereas poor diagnostic accuracy is indicated by AUC values as low as 0.5. The comparison among AUCs was evaluated using logistic regression analysis using MedCalc 14.8.1 (MedCalc Software bvba). The ROC curve was generated using SPSS 22.0 package. A P-value of <0.05 was considered statistically significant.

## 3. Results

### 3.1. Basic information of participants

The 205 lung cancer patients were enrolled in this study. There are 152 males and 53 females, whose mean age was 61.45 ± 10.52 (33–87 years). In addition, another 186 lung benign disease patients were also enrolled in this study, including 113 males and 73 females, whose mean age was 62.98 ± 15.01 (19–98 years). The demographic characteristics of lung cancer and control patients are shown in Table 1.

### 3.2. The Hsp90α level in patients

**Figure 1:**
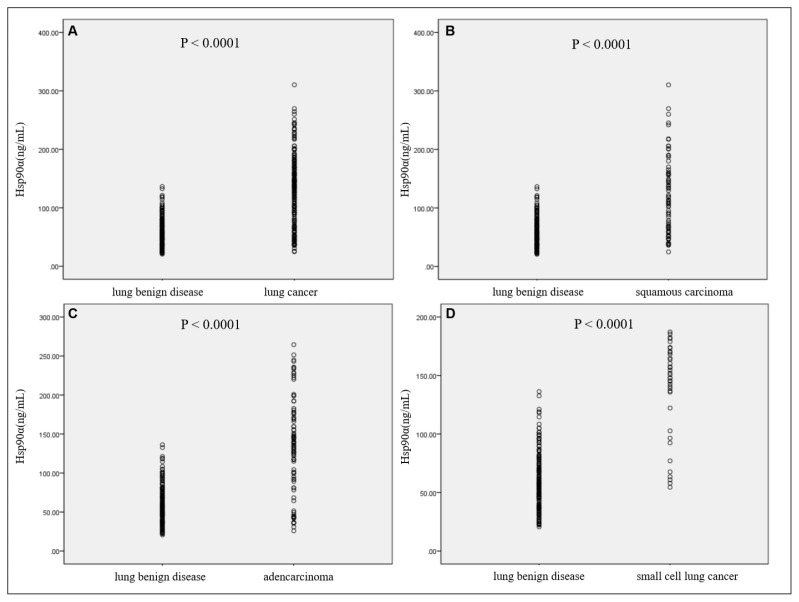
The serum levels of Hsp90α in patients with lung cancer (A), squamous cell carcinoma (B), adenocarcinoma (C), and small cell lung cancer (D) compared with benign lung diseases.

Compared with benign lung disease patients, the serum level of Hsp90α (128.23 ng/mL, ranging from 76.40 to 167.20 ng/mL) in lung cancer patients was higher (p < 0.0001, Figure 1A).

We investigated the Hsp90α level in three pathological types of lung cancer. As shown in Figures 1B, 1C, and 1D, compared with benign lung disease patients, the Hsp90α level was significantly higher in squamous cell carcinoma, adenocarcinoma, and small-cell lung cancer. According to the results of statistical analysis, we did not observe a statistically significant difference for plasma Hsp90α levels compared according to age, gender, three pathological cell type, smoking status, metastasis condition except TNM stage (Table 2).

### 3.3. Diagnostic value of Hsp90α in lung cancer

**Figure 2:**
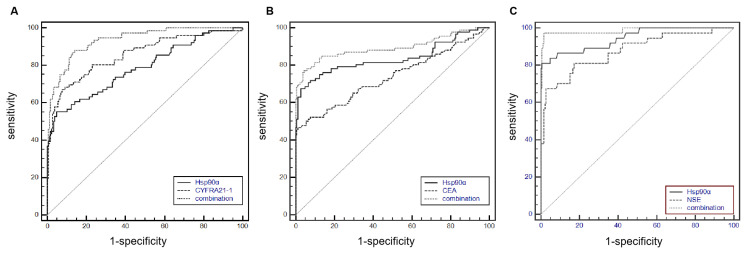
ROC curves of Hsp90α, CYFRA21-1, and the combination of these factors in the diagnosis of patients with squamous cell carcinoma and lung benign disease (A), ROC curves of Hsp90α, CEA, and the combination of these factors in the diagnosis of patients with carcinoma and lung benign disease (B), ROC curves of Hsp90α, NSE, and the combination of these markers in the diagnosis of patients with small cell lung cancer and small cell lung cancer (C).

The 205 cases of lung cancer were divided into squamous cell carcinoma (76 cases), adenocarcinoma (92 cases), and small cell lung cancer (37 cases). In 76 cases of squamous cell carcinoma, as shown in Figure 2A and Table 3, the AUC of Hsp90α combined with CYFRA21-1 was significantly higher than that of CYFRA21-1 alone (0.937, 95% CI: 0.900–0.963 vs. 0.858, 95% CI: 0.809–0.898, p = 0.0049). The PPV and NPV of biomarker combination (CYFRA21-1+Hsp90α) were 0.913 (0.850–0.951) and 0.813 (0.752–0.863). Meanwhile, compared with CEA or NSE alone, the AUC of Hsp90α combined with CEA or NSE was significantly higher in the diagnosis of adenocarcinoma or small-cell lung cancer. The PPV and NPV of biomarker combination (CEA+Hsp90α) were 0.813 (0.746–0.863) and 0.946 (0.898–0.972), respectively. The PPV and NPV of biomarker combination (NSE+Hsp90α) were 0.980 (0.943–0.993) and 0.970 (0.854–0.994), respectively (Figures 2B and 2C, Table 4–5).

### 3.4. The dynamic changes of Hsp90α in response to chemotherapy

**Figure 3:**
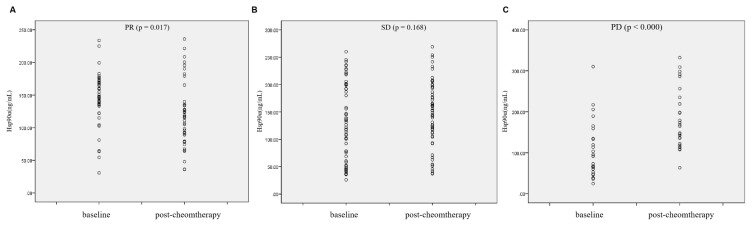
Patients with advanced lung cancer were grouped into PR (A), PD (B), and SD (C) according to treatment response. The serum levels of Hsp90α were reassessed after the second cycle of chemotherapy.

We further measured serum Hsp90α levels in 127 advanced lung cancer patients who received chemotherapy. The results showed that the Hsp90α level was significantly decreased after chemotherapy in the PR group (119.48 ± 7.63 vs. 143.71 ± 6.41 ng/mL, n = 42, p = 0.017, Figure 3A). Hsp90α level was not significantly changed after treatment in SD group (147.12 ± 7.75 vs. 130.63 ± 9.01 ng/mL, n = 59, p = 0.168, Figure 3B). However, Hsp90α was increased after chemotherapy treatment in the PD groups (183.31 ± 14.46 vs. 104.65 ± 13.54 ng/mL, n = 26, p < 0.000, Figure 3C).

## 4. Discussion

To improve the diagnosis of lung cancer, many tumor markers, including CYFRA21-1, CEA, and NSE, have been intensively evaluated and have been widely used in the diagnosis of lung cancer. However, each marker has its own specificity and sensitivity, which might lead to limitations in the diagnosis. The combined detection of tumor markers may be of great importance in the diagnosis of tumors. Hsp90α has also been demonstrated to have diagnostic value in lung cancer. In the present study, we demonstrated that the detection of Hsp90α combined with that of CYFRA21-1, CEA, and NSE could significantly increase the AUC and the sensitivity.

Hsp90α is a chaperone protein that regulates protein folding. It also helps proteins remain stable in response to oxidative and heat stress, as well as protein degradation processes [11]. Hsp90α is considered as a tumor marker for the diagnosis of colorectal cancer, prostate, etc [12–14]. As reported by Shi et al. [15], the Hsp90α level was increased in advanced lung cancer patients. In the present study, Hsp90α was significantly higher in patients with lung cancer compared to those with benign lung disease. It was also higher in advanced lung cancer (III–IV stage) than in early-stage (I–II stage) cancer, which indicated that Hsp90α may play an important role in the progression of lung cancer.

ROC analysis has been used extensively to compare the diagnostic value of tumor markers. The AUC is considered a quantitative measure of the discrimination power of tumor markers in the differentiation of lung cancer cases from benign lung diseases. CYFRA21-1, CEA, NSE, and other tumor markers are widely used in the diagnosis of lung cancer. As they have their own specificity and sensitivity, hence they have different diagnostic potential. Therefore, combining different markers may improve the diagnostic value of individual markers [16]. In the current study, we found that Hsp90α combined with CYFRA21-1, CEA, and NSE can improve the diagnostic level of lung cancer, which was consistent with the study of Shi et al. [15]. In addition, lung cancer patients were further divided into subgroups, squamous cell carcinoma (Table 3), lung adenocarcinoma (Table 4), and small cell lung cancer (Table 5). CYFRA21-1, a fragment of cytokeratin 19, is mainly expressed in tumor cells of epithelial origin and can be used as a marker for epithelial cancers. According to one study, the level of CYFRA21-1 in squamous cell carcinoma was higher than that in adenocarcinoma and SCLC [16]. In the present study, the sensitivity and specificity of squamous cell carcinoma by CYFRA21-1 were 0.671 and 0.830, respectively. When CYFRA21-1 was combined with Hsp90α, the sensitivity and specificity of the combination were both increased. Meanwhile, the sensitivity and specificity of lung adenocarcinoma by CEA were 0.467 and 0.929, respectively. When CEA was combined with Hsp90α, the sensitivity and specificity of the combination were also both increased. Moreover, the sensitivity and specificity of lung adenocarcinoma by NSE were 0.757 and 0.880, respectively. When NSE was combined with Hsp90α, the sensitivity and specificity of the combination were also both increased. These combinations provided good diagnostic accuracy for carcinoma and small cell lung cancer, which suggested that Hsp90α combined with these markers provided a better diagnostic value for lung cancer.

Furthermore, we assessed the value of Hsp90α in predicting the response of patients with lung cancer to chemotherapy. Results showed that the Hsp90α level was higher in the PD group compared to SD and PR groups. Consistently, Žáčková et al. demonstrated that lower serum levels of Hsp90α in patients with chronic myeloid leukemia were correlated with good response to therapy [17]. Collectively, our results suggested that Hsp90α may be used to monitor therapeutic responses in patients with lung cancer.

Moreover, we show that Hsp90α combined with CYFRA21-1, CEA, and NSE have a higher diagnostic value in lung cancer. Furthermore, Hsp90α measurement may help monitor treatment response. However, several limitations in this study are worth mentioning, such as the retrospective nature of the study and the small number of patients for each lung cancer subtype.

## Figures and Tables

**Table 1:** The demographic characteristics of lung cancer and benign lung disease.

Group	lung cancer (n = 205)	benign lung diseases (n = 186)
Age (years)	61.45 ± 10.52 (33–87)	62.98 ± 15.01 (19–98)
Gender (male/female)	152/53	113/73
BMI	26.34 ± 5.54 (17.01–38.12)	24.78 ± 5.42 (16.24–36.97)
Smoking or former smoking	92	79
Comorbidities (cases)		
Diabetes mellitus	23	27
Hypertension	56	41
Chronic gastropathy	21	18
Cerebrovascular diseases	34	37

**Table 2:** Relationships between the Hsp90α concentration and clinic-pathologic characteristics in 205 lung cancer patients. Data were presented as Median (P_25_–P_75_).

Items	Cases (n)	Hsp90α (ng/mL)	P-value
Gender			0.586
Male	152	129.59 (82.01–169.75)	
Female	53	124.35 (62.48–156.57)	
Age, year			0.926
<60	80	128.07 (79.14–160.53)	
≥60	125	128.03 (70.87–169.71)	
Smoking status			
Never	113	121.39 (65.26–159.98)	0.070
Smoking or former smoking	92	136.64 (102.92–172.58)	
Pathological type			0.146
Squamous	76	117.79 (60.17–158.79)	
Adenocarcinoma	92	132.90 (91.79–170.93)	
Small cell lung cancer	37	138.07 (102.47–169.02)	
TNM stage			0.044
I+II	49	113.16 (62.81–151.81)	
III+IV	156	132.97 (78.28–173.88)	
Metastasis condition			0.406
Brain metastasis	21	119.61 (60.55–166.90)	
Bone metastasis	11	132.03 (60.57–180.12)	
Pleural metastasis	40	144.82 (104.55–197.62)	
Multiple metastases	64	140.09 (107.27–168.60)	

**Table 3:** The ROC of the conjoint analysis of CYFRA21-1 and Hsp90α in lung squamous cell carcinoma.

Items	CYFRA21-1	Hsp90α	CYFRA21-1 +Hsp90α
Optimal cut-off	4.77 (ng/mL)	101.78 (ng/mL)	-
Sensitivity	0.671	0.553	0.882
Specificity	0.830	0.952	0.860
Younden’s index	0.596	0.505	0.742
SE	0.0279	0.0339	0.0516
AUC (95% CI)	0.858 (0.809–0.898)	0.787 (0.732–0.835)	0.937 (0.900–0.963)
NPV (95% CI)	-	-	0.913 (0.850–0.951)
PPV (95% CI)	-	-	0.813 (0.752–0.863)
P-value*	0.0049	<0.001	-

* Comparing the area under ROC curve of combination to single tumor marker in lung squamous cell carcinoma vs. benign lung disease group.

**Table 4:** The ROC of the conjoint analysis of CEA and Hsp90α in lung adenocarcinoma.

Items	CEA	Hsp90α	CEA+Hsp90α
Optimal cut-off	5.7 (ng/mL)	114.78 (ng/mL)	-
Sensitivity	0.467	0.685	0.772
Specificity	0.929	0.973	0.957
Younden’s index	0.456	0.658	0.729
SE	0.0358	0.0255	0.0311
AUC (95% CI)	0.737 (0.681–0.788)	0.837 (0.789–0.879)	0.893 (0.851–0.927)
NPV (95% CI)	-	-	0.813 (0.746–0.863)
PPV (95% CI)	-	-	0.946 (0.898–0.972)
P-value*	<0.001	0.0095	-

* Comparing the area under ROC curve of combination to single tumor marker in lung adenocarcinoma vs. benign lung disease group.

**Table 5:** The ROC of the conjoint analysis of NSE and Hsp90α in lung small cell lung cancer.

Items	NSE	Hsp90α	NSE +Hsp90α
Optimal cut-off	16.325 (ng/mL)	102.95 (ng/mL)	-
Sensitivity	0.757	0.703	0.919
Specificity	0.880	0.960	0.993
Younden’s index	0.637	0.663	0.912
SE	0.0362	0.0230	0.0116
AUC (95% CI)	0.877 (0.827–0.917)	0.945 (0.906–0.979)	0.986 (0.961–0.997)
NPV (95% CI)	-	-	0.980 (0.943–0.993)
PPV (95% CI)	-	-	0.970 (0.854–0.994)
P-value*	0.009	0.044	-

* Comparing the area under ROC curve of combination to single tumor marker in lung small cell lung cancer vs. benign lung disease group.
